# Study protocol of a randomised controlled trial to examine the impact of a complex intervention in pre-frail older adults

**DOI:** 10.1007/s40520-018-1106-7

**Published:** 2019-01-02

**Authors:** Ruth Teh, Ngaire Kerse, Debra L. Waters, Leigh Hale, Avinesh Pillai, Evelingi Leilua, Esther Tay, Anna Rolleston, Richard Edlin, Eruera Maxted, Claire Heppenstall, Martin J. Connolly

**Affiliations:** 1 Department of General Practice and Primary Health Care, School of Population Health, University of Auckland, Auckland, New Zealand; 2 School of Population Health, University of Auckland, Auckland, New Zealand; 3 Department of Medicine, School of Physiotherapy, University of Otago, Dunedin, New Zealand; 4 Centre for Health, Activity and Rehabilitation Research, School of Physiotherapy, University of Otago, Dunedin, New Zealand; 5 Department of Statistics, Faculty of Science, University of Auckland, Auckland, New Zealand; 6 The Centre of Health, Tauranga, New Zealand; 7 Health Systems Group, School of Population Health, University of Auckland, Auckland, New Zealand; 8 Lakes District Health Board, Rotorua, New Zealand; 9 Department of Medicine, University of Otago, Christchurch, New Zealand; 10 Department of Geriatric Medicine, University of Auckland, Auckland, New Zealand; 11 Waitemata District Health Board, Auckland, New Zealand

**Keywords:** Frailty, Nutrition, Physical activity, Older adults, Cost effectiveness

## Abstract

**Background:**

Frailty is a multidimensional geriatric syndrome associated with functional loss. The Senior Chef (SC, nutrition) and SAYGO (strength and balance exercise) programmes are well accepted among older adults but the impact of each, or a combination of both, on the frailty syndrome in pre-frail older adults is unknown.

**Aims:**

To determine the effectiveness and cost-effectiveness of a complex intervention consisting of the SC and/or SAYGO programmes to prevent progression of frailty in pre-frail older adults.

**Methods:**

A multi-centre randomised controlled assessor-blinded study. The four intervention groups are SC, an 8-week nutrition education and cooking class; SAYGO, a 10-week strength and balance exercise class; SC plus SAYGO, and a social group (Control). Community-dwelling adults aged 75+ (60 + Māori and Pasifika) in New Zealand are recruited through health providers. Participants are not terminally ill or with advanced dementia, and have a score of 1 or 2 on the FRAIL questionnaire. Baseline assessments are completed using standardised questionnaires prior to randomisation. Four follow-up assessments are completed: immediately after intervention, 6, 12 and 24 months post-intervention. The primary outcome is frailty score, secondary outcomes are falls, physical function, quality of life, food intake, physical activity, and sustainability of the strategy. Study outcomes will be analysed using intention-to-treat approach. Cost analyses will be completed to determine if interventions are cost effective relative to the control group.

**Discussion:**

This trial is designed to be a real world rigorous assessment of whether the two intervention strategies can prevent progression of frailty in older people. If successful, this will generate valuable information about effectiveness of this nutrition and exercise strategy, and provide insights for their implementation.

**Trial registration:**

Australian and New Zealand Clinical Trials Registry number—ACTRN12614000827639.

## Background

Frailty is a precursor to functional loss [1]. It is a multidimensional geriatric syndrome involving several inter-related physiological systems described as a reduction in reserve to meet homeostatic challenge, meaning frail older people are vulnerable to poor outcomes [2] including increased risk of falls, impaired cognitive function, worsening disability, hospitalisation, residential care admissions and mortality [3]. Up to 17% of community-dwelling older people aged 65 + have been reported as frail with 42% pre-frail [3, 4]. In octogenarians, over 60% have been reported to be pre-frail [5].

In the pre-frail state, an older person has homeostatic mechanisms to respond to external stressors, but less than those who are not frail. This transition phase may provide a window of opportunity to prevent deterioration of health outcomes and to maintain quality of life.

Several strategies have attempted to reduce frailty to improve outcomes, with physical activity and nutrition being the most commonly tested interventions [6]; however, results are inconsistent and a greater focus on prevention is needed to reduce the poor outcomes associated with frailty.

Older people are vulnerable to poor nutritional status [7] and inadequate food intake is related to pre-frailty and frailty [8]. Supplements increase weight, particularly in those with malnutrition [9] but the impact of supplements on frailty is uncertain and their wider implementation may be costly. In the InCHIANTI study, 700 + older people, mean age 73 years, adherence to a healthier dietary pattern was associated with a lower risk of being frail over 6-year follow-up [10]. Self-preparation of meals stimulates appetite and is associated with intake of more nutritious meals [11]. Cooking also involves physical and social skills (e.g. shopping for ingredients) and cognitive abilities [11]. Senior Chef (SC) is a programme developed in New Zealand to increase knowledge and practical skills in shopping, cooking and general nutrition for older people. The programme has been evaluated [12] but impact on health outcomes is uncertain.

Physical inactivity increases with age [13]. In New Zealand, after the age of 75 years, approximately 30% men and 40% women were physically inactive, i.e. doing less than 30 min of physical activity per week [14]. Studies in older adults have shown that physical activity interventions improve participation in ‘life’ [15] and this may be through improved physical function, including better flexibility, and cardiorespiratory and muscle fitness particularly in frail older adults [16]. Physical activity trials have had some success in changing lifestyle [17–20] but there is no widespread implementation of programmes for frail and pre-frail older people in New Zealand, with Māori neglected almost completely. Exercise needs to be sustainable for ongoing benefit. The Steady As You Go (SAYGO) programme is a group-based exercise, and was developed from the Otago Exercise Programme set of exercises that has been proven to prevent falls [21]. SAYGO uses a peer-led model, with the group led by a trained facilitator and then transitioning to peer leadership, a model shown to be acceptable and sustainable [22]. The SAYGO programme has been shown to decrease injurious falls [23] and enhance social capital in older adults [24].

According to the Fried criteria, the frailty phenotype includes weight loss, poor endurance and energy, slowness, weakness and low physical activity [25]. Energy and nutrient intake must balance physical activity to maintain a neutral energy expenditure to maintain weight. Furthermore, adequate nutrients are essential for musculoskeletal function. Integrating nutrition and physical activity may be more important in frail/pre-frail than robust older adults to restore an optimal homeostatic mechanism.

Considering the nutrition and exercise focus of the SC and SAYGO programmes, respectively, these have the potential to reverse or slow the progression of frailty in pre-frail older adults using interventions that are acceptable to older adults. Sustainability of these programmes is not only driven by peer leaders but also the availability of funding. For older persons, and health care and service providers, the “value for money” of the intervention is the main interest. For healthcare funders, it is a priority to explore avenues to reduce healthcare costs with the ageing population. The effectiveness and cost-effectiveness the SC and SAYGO programmes to prevent frailty will be determined in this intervention study.

## Methods

This trial aims to determine the effectiveness and cost-effectiveness of the SC and/or SAYGO programmes to prevent frailty in pre-frail older adults.

Design: A multi-centre randomised controlled, assessor-blinded, 2 × 2 factorial study.

Setting and characteristics of participants: Community-dwelling older adults aged 75+ (60 + Māori and Pasifika) across four sites in both the main islands of New Zealand (North and South Islands), with no upper age limit. Māori are the indigenous Polynesian people of New Zealand and Pasifika people are migrants from the South Pacific Region who are living in New Zealand. Health inequalities between the Māori/Pasifika and non-Māori/non-Pasifika are evident [26] and Māori/Pasifika people have lower life expectancy than non-Māori/Pasifika [27], so that applying uniform eligibility criteria would exclude Māori and Pasifika older people.

### Recruitment

Participants are recruited by a mailed invitation signed by the general practitioner (GP) or through Māori health providers (who deliver health and disability services using a cultural framework distinctive to Māori people) by phone or personal invitation from the provider. In New Zealand, 93% of the population are enrolled with a general practice. Practices and providers peruse a list of all enrolled age and ethnicity eligible patients prior to the postal invitation. Eligibility criteria includes not being terminally ill or having advanced dementia as judged by the GP or Māori health provider, living in the community, able to stand, medically safe to participate in low-intensity exercise, and able to use the kitchen utensils safely. Pre-frail status is ascertained using the FRAIL questionnaire [28, 29] (by a score of 1 or 2). The postal pack including the invitation letter and study information along with the FRAIL questionnaire, a reply card, and a reply prepaid envelope is sent to potential participants to facilitate interest and willingness to participate. Those not returning the reply card are contacted by research staff in practices comfortable with this contact for their patients. Eligible older adults giving written informed consent are enrolled in the study. The study was approved by the Southern Health and Disability Ethics Committee, Ministry of Health, New Zealand (Ref 14/STH/101/, 13th August 2014).

### Interventions

After participants complete baseline assessments, they are randomly assigned to one of four groups: SC, SAYGO a group strength and balancing exercise, both SC and SAYGO, and social group (control group).

**Senior Chef** is a weekly 3-h session run for 8 weeks by a trained facilitator following set lesson plans. The 3-h session comprises a morning tea (or light snack) prepared by the facilitator, nutrition education, cooking together (hands-on cooking in pairs) using provided utensils and cooking facilities and then sharing the meal cooked that day. The nutrition education covers practical information about sourcing food, menu planning, budgeting and shopping tips and written information is provided to attendees to take home. After completion of the 8-week programme, the group is encouraged to continue to meet as peers as coordinated by the facilitators.

SAYGO (Steady As You Go) is a weekly 1-h session of exercises to strengthen lower limb muscles and to challenge balance for 10 weeks led by a professionally trained facilitator. The exercises are based on the Otago Exercise Programme, one proven to reduce falls and be sustainable [30]. The programme was adapted for group delivery by physiotherapists and found to be effective in improving physical function in those who participate [22]. During the first 6 weeks, potential peer leader(s) are encouraged by the professionally trained facilitator to consider being a peer leader. Other participants in the group are encouraged to support the peer-led class by helping arrange and set up the venue, collect gold coin donations and take attendance. At week 9, peer leaders are invited to take the class in their community and go through a 1-day training. The training includes a physiotherapy-developed manual and covers class safety, fall prevention, specific exercise routines and first aid workshop. There is a CD with all the exercises that peer leaders use to teach their class to maintain fidelity. The peer leaders and class members decide on the venue for the classes, and continue to meet weekly.

Social group (control) is a weekly 1–2-h “seated” (i.e. non-physical in nature) socialising group facilitated in the local community for 10 weeks. Following the 10-week period, participants are encouraged to continue to attend social groups.

In the trial, attendance sheets are maintained throughout the programmes (by the trained facilitators) and subsequent classes (by the facilitators or peer leaders); no exercise diary or daily dietary intake will be recorded. Transport arrangement is facilitated or provided (if required). Where possible, the facilitators encourage shared rides and source available low-cost travel.

### Outcome measures

Primary outcome: Changes in frailty score. The definition of frailty, adapted from Fried et al. [25] is defined as the presence of three or more of the following characteristics: weight loss, weak grip strength, poor endurance/exhaustion, low physical activity, and slow gait speed.

- Weight is measured using Tanita BC-545N scale to the nearest 0.1 kg. Those with ≥ 5% of weight loss in the prior year (by direct measurement of weight) are considered to have weight loss. Weight loss at baseline is determined using the question from FRAIL questionnaire “Have you lost weight in the past 12 months without trying to?” A response of “Yes and loss more than 3 kg/0.5 stones/7 pounds” is considered to indicate ‘weight loss’.
- Grip strength is determined using the Takei digital handgrip dynamometer Grip D on both hands in a standing position. A grip strength of < 30 kg for men and < 20 kg for women is considered as having weakness [31].
- Gait speed is determined using the 3-m walk measured with a measuring tape and timed with a stopwatch, conducted in a place with enough space to ensure at least 1 m before and after the 3-m timed allotment. A cut-off of < 0.8 ms^−1^ is considered as having a slow gait speed [32].
- Exhaustion is determined using a question from the FRAIL questionnaire “How much of the time during the past 4 weeks did you feel tired?” 1 = all of the time, 2 = most of the time, 3 = some of the time, 4 = a little of the time, and 5 = none of the time. Responses of “1” or “2” are considered as having exhaustion, and all others as not [33].
- Physical activity is determined using the Community Healthy Activities Model Program for Seniors—CHAMPS Activities Questionnaire [34, 35]. Those who reported less than 150 min of moderate intensity activity per week on the CHAMPS questionnaire are considered to have a low physical activity. This estimate is derived from the New Zealand Ministry of Health current physical activity recommendations for older adults of at least 30 min of aerobic physical activity for 5 days each week [14].

Secondary outcomes: falls and physical function

A fall will be defined as “an unexpected event in which the participant comes to rest on the ground, floor, or lower level”, as per guidelines published by the Prevention of Falls Network Europe (ProFaNE) [36]. In ascertaining falls at baseline, participants will be asked “In the previous 12 months, have you had any fall including a slip or trip in which you lost your balance and landed on the floor or ground or lower level?” Falls will be recorded prospectively by the participant using a fall calendar where the participant marks daily if they had a fall. The monthly fall calendar will be returned to the University of Auckland in provided prepaid postage envelopes. Telephone interview by an interviewer blinded to group allocation will be carried out to ascertain further details of falls and injuries, and to follow-up with participants if the monthly fall calendar is not received or to rectify missing data. A blinded medically trained assessor will examine all fall reports to ensure a defined fall has occurred and establish the presence of injury. Injury will be categorised as absent if none recorded, mild to moderate for report of pain, inability to perform usual activities after the fall, bruising, and contusions. Severe injury is any fracture (excluding finger fracture) or any admission to hospital overnight. Rate of falls, risk of falls, and time to first fall will be reported as recommended by Lamb et al. [36].

The validated short physical performance battery (SPPB) will assess physical performance [37]. The SPPB is an objective assessment tool for evaluating lower extremity functioning in older persons. It consists of three parts: the Balance Test, the Gait Speed Test, and the Chair Stand Test, and it can be administered in approximately 10 min. It has a score between 0 and 12 points, the higher the score the better the physical performance. The SPPB is a widely used measure of physical performance for it is practical and safe to be administered by trained assessors in home setting [38].

Functional status will be assessed using the Nottingham Extended Activities of Daily Living (NEADL), a commonly used instrument for assessing activities of daily living within four categories: mobility, kitchen activities, domestic activities and leisure activities. It has a score between 0 and 22, the higher the score the better the functional status [39, 40].

Health-related quality of life will be assessed with the Short Form (SF-12) including the summary scores for physical and mental health-related QOL [41]. Individual-level responses from the SF-12 can be translated into a preference-based quality of life measuring health on a utility scale where ‘dead’ scores 0 and ‘full health’ scores 1. These repeated individual utilities can be used to construct QALYS [41] by plotting time against quality of life and identifying the area under the plotted curve. This QALY measure identifies health over a period of time and is used in the cost-effectiveness analysis.

Process outcomes will also be used as secondary outcomes, namely food intake and reduced sedentary time. Food intake will be assessed using the INTAKE24, an online dietary tool developed by a team in Newcastle University, Newcastle upon Tyne, UK [42]. INTAKE24 is based on the 24-h multiple-pass recalls (MPR) dietary assessment with the New Zealand FOODfiles 2016 database incorporated into the programme. The MPR protocol is suitable for use in the general [43] and oldest old population and matches that used in the Newcastle 85+ and LiLACS NZ studies [44, 45]. Two 24-h MPR will be completed on two separate days (a weekday and a weekend) by a trained interviewer at the participants’ residence or a venue suitable for them.

Physical activity will be determined using the CHAMPS Activities Questionnaire. CHAMPS is a validated questionnaire to measure sedentary (1 METs), low-light (> 1 and ≤ 2 METs), high-light (> 2 and < 3 METs), moderate-to-vigorous physical activity (MVPA, ≥ 3 METs), and total activity in older people over a 4-week period [34, 35].

### Covariates

Standardised questionnaires will be administered with a face-to-face interview by trained interviewers. Data collected will include socio-demographic (living arrangement, marital status, education, lifetime occupation), smoking, alcohol consumption, medical history, use of medications (prescribed and non-prescribed), hearing and visual impairment, falls, fear of falling (assessed with the Fall Efficacy Scale-International, FES-I) [46], cognitive function (assessed using the Montreal Cognitive Assessment, MoCA) [47], depressive symptoms (Geriatric Depression Scale, GDS) [48], anthropometric measures (height, weight, body composition (Tanita Scale BC 545N), circumference of the waist, hip, calf and mid arm), and blood pressure (Omron HEM7130).

### Participant time

**Fig. 1:**
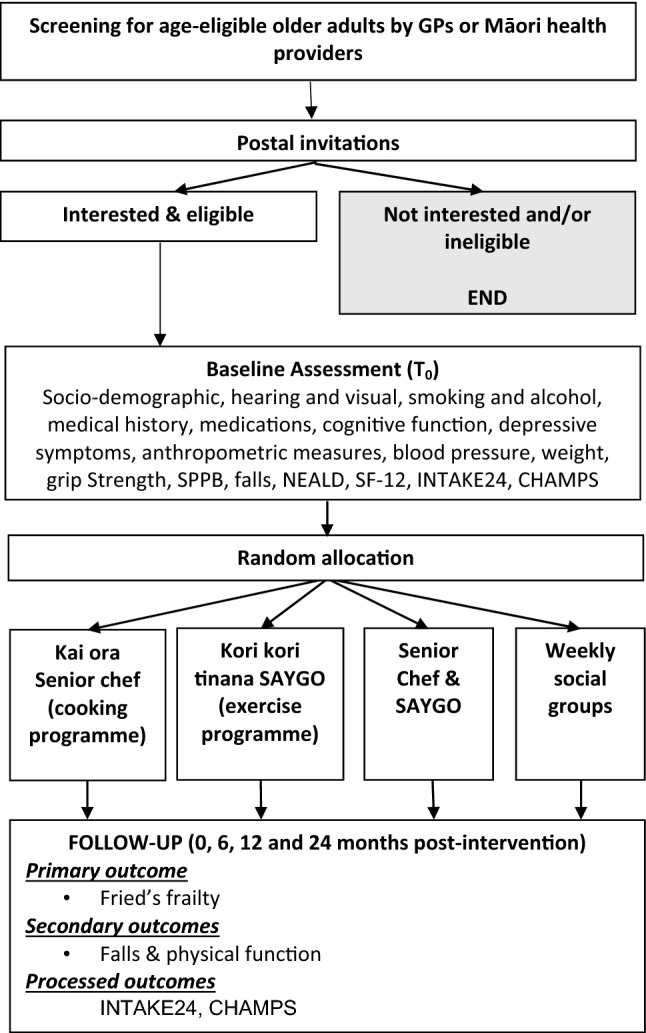
Study flowchart

Figure 1 below depicts the recruitment process and assessment schedule. Table 1 shows the detailed follow-up assessments.

**Table 1:** Assessment schedule

Time point	Study period							
	Enrolment	Allocation	Post-allocation					Close-out
	*t*_0_ (baseline)	0	*t*_intv_	*t*_1_ (0 m)	*t*_2_ (6 m)	*t*_3_ (12 m)	*t*_4_ (24 m)	*t*_x_
Enrolment								
Eligibility screen	X							
Informed consent	X							
Random allocation		X						
Interventions								
Senior chef			X					
SAYGO			X					
SC & SAYGO			X					
Social			X					
Assessments								
Socio-demographic	X							
Hearing and visual	X						X	
Smoking and alcohol	X						X	
Medical history	X					X	X	
Medications	X					X	X	
Cognitive function	X				X	X	X	
Depressive symptoms	X			X	X	X	X	
Anthropometric measures & blood pressure	X			X	X	X	X	
Outcome measures								
Weight	X			X	X	X	X	
Grip strength	X			X	X	X	X	
SPPB	X			X	X	X	X	
Falls	X			X	X	X	X	
NEALD	X			X	X	X	X	
SF-12	X			X	X	X	X	
INTAKE24	X			X	X	X	X	
CHAMPS	X			X	X	X	X	
Dissemination								
Back to participants, GPs, local health professionals								X
Academic community								X

*CHAMPS* Community Healthy Activities Model Program for Seniors, *GP* general practitioners, *NEADL* Nottingham Extended Activity of Daily Living, *SPPB* Short Physical Performance Battery

### Sample size

The sample size and power calculations are based on the primary outcome of frailty score. A sample of 504 (126 in each of group) will provide 90% power at a 5% significance level (two sided) to detect a 25% reduction in the mean number of frailty criteria (reduction of 0.43 from 1.68 to 1.25) [49]. With an attrition rate of 20%, the study will enrol 600 eligible older people. This sample size will also provide 80% power at a 5% significance (two sided) to detect a 30% reduction in the rate of falls, reduction of 30% [50] in falls (from 58 to 41% [51]).

### Allocation

The randomisation sequence will be prepared by an independent statistician using computer-generated block randomisation and uploaded to the Electronic Data Capture (EDC) system by an administrator of the EDC. The randomisation sequence was stratified by study sites (Whangarei, Howick-Auckland, Tauranga, Invercargill) and ethnic groups (Māori/Pasifika and non-Māori/Pasifika). The allocation sequence will be concealed to all study investigators and the study coordinators involved in enrolment. Local study coordinators will assign the intervention group through the EDC after the assessors complete baseline assessments. Once randomisation has occurred, the study coordinators and participants are not blinded to group allocation. The study coordinators systematically remind all participants not to disclose the group allocation to the assessors prior to the follow-up appointments.

### Blinding

The trained research assessors will collect all baseline measurements of primary and secondary outcomes prior to randomisation. The same assessors blinded to the group allocation will conduct follow-up assessments. Assessors will be asked of their ‘blinding’ status at every assessment time point.

Fall calendars will be completed by the participants and sent to the central study coordinating office based at the University of Auckland. A blinded assessor will interview participants on the phone about the falls that are reported or to check missing data or missed submission of calendars.

All investigators will be blinded to the treatment allocation to minimise any potential influence on the progress and outcome measures except for one appointed investigator who will work closely with the site coordinators and will not be blinded to the group allocation.

### Data collection, management, and analysis

Data will be collected with face-to-face interviews and physical assessments using a standardised questionnaire completed by trained research assessors blinded to the group status of the participant in the participant’s home or local clinic. Data will be entered directly to the EDC system. Assessors are trained over a 2-day training period for all measures including standardisation. Assessors will meet regularly to support each other and observe each other’s procedures. Research-trained assessors blinded to group status will complete covariate and outcome assessments at baseline, end of the intervention (0 month  post-intervention), 6 months, 12 months and 24 months post-intervention.

Data entered in the EDC will be verified for completeness by the data quality assurance research team (see “Monitoring” section). Data from the EDC will be transferred to a statistical package (SAS and SPSS) for analysis. INTAKE 24 data are automatically linked to NZ FOODfiles and macro- and micronutrients are calculated.

The primary approach for analysis of primary and secondary outcomes will follow the intention-to-treat principle, for all participants who have at least one baseline measurement. All randomised participants will be analysed in the group they were allocated to, even if they do not attend the allocated intervention programme, are lost to follow-up, or die thereby preserving the intention-to-treat framework. The primary approach for analyses will be supplemented by a secondary analysis which will be a per-protocol analysis of outcomes for those who participated in the interventions and includes only observed cases (i.e. participants with measurements at baseline to the 24 months).

### Statistical methods

Descriptive statistics will be used to summarise information collected on each group statistics. Statistical significance will be assumed at *p* < 0.05.

#### Primary analysis method

A repeated measures generalised linear model will be used to compare the mean number of frailty criteria between randomisation groups over time. The effect of potential confounders (age, number of co-morbidities, number of prescribed medication, cardiovascular health status, energy intake) will be adjusted for in the regression models.

#### Secondary analysis methods

Fall data will be summarised as number of falls, number of fallers/non-fallers/frequent fallers, fall rate per person year, and time to first fall.

The absolute risk difference between groups will be reported. We will use negative binomial regression to estimate the difference in fall rates between groups. The effect of potential confounders (number of co-morbidities, number of prescribed medication, use of psychotropic medications and cardiovascular health status) will be adjusted for in the regression models. Interactions between falls and frailty (overall and separately for gender and ethnicity) will be examined.

For outcomes with baseline and the post-intervention 24-month follow-up measures only, comparisons of continuous variables between groups will be performed using *t* tests. Where the continuous variables are not normally distributed, non-parametric comparisons using the Wilcoxon rank-sum test will be used. Categorical measures will be compared using the Chi-square test. For outcomes with repeated measures (more than two measurements), generalised estimating equations (GEE) will be used with clustering on participant and an exchangeable correlation matrix.

#### Handling of missing data and loss to follow-up

The data will be checked for missing values. If there are few participants with missing values (< 1%) for many important covariates (such as physical assessment e.g. weight and grip strength), they will be removed from the analysis of primary and secondary outcomes. If there is less than 1% of data missing then no imputation will be carried out. A sensitivity analysis will examine whether including or excluding imputation makes a difference to the result. Every effort will be made to minimise missing data. Secondary analyses (limited to the primary outcome) will be performed that involve joint modelling of missing data for sensitivity assessment. The statistical packages SAS 9.4 and IBM SPSS Statistics 25 will be used for all analyses.

*Cost-effectiveness analysis* will consider whether either or both interventions are cost effective relative to social contact (no intervention) and will (initially) report on a within-trial basis from within-a-payer (District Health Board) perspective. Costs counted include intervention costs, residential care costs, hospitalisation costs (from routinely collected New Zealand Ministry of Health data), and will be valued according to the type of hospital admission (e.g. diagnosis/speciality) and length of stay. Unit costs will be sourced where available from national level data, supplemented by information from the literature where no suitable national level source exists. Quality of life is assessed from baseline to 12 months post-intervention and will be used to estimate quality-adjusted life years (QALYs), with the value for money of the intervention assessed using incremental cost-effectiveness ratios. As the analysis is within 12 months, discounting is not required and the analysis will use 2017/18 New Zealand Dollars as its base year.

### Monitoring

Study sites are visited by the Principal Investigator or a designee on at least 6-monthly basis. Study progress will be discussed with the site coordinators and study documents (i.e. consent forms, recruitment logs, attendance list) are checked for completeness and accuracy. Interim checks on progress are made by telephone when deemed appropriate. Refresher training is completed annually by the PI with the blinded assessors for fidelity of the assessment procedures.

For data validation, one member of the central study coordinating office at the University of Auckland or its designee will inspect the recruitment log and compare them with the source data, i.e. the FRAIL screening tool and signed informed consent form. Data completeness on the EDC is checked by a second designated member of the central study coordinating office within 4 weeks of completion of the interview and queries are rectified by the assessors within 2 weeks.

The Data and Safety Monitoring Board (DSMB) comprises a statistician and an experienced clinical trialist, neither who have involvement with the day-to-day running of the study. The DSMB will be responsible for reviewing any matters of safety that arise during the course of the study.

### Participant consent

The consent form was approved by the Southern Health and Disability Ethics Committee, Ministry of Health, New Zealand, prior to the beginning of the trial. The site coordinator will visit an older person who is eligible and go through the participant information sheet and address any questions that arise. If the participant is unable to read, an impartial witness will be present during the entire discussion and will be responsible for signing and dating the form on the participants’ behalf if he or she is unable to write in English. Ample time will be provided to the participant, accompanying person or legal representative to enquire about the trial and decide whether to participate. Participant written consent will be obtained prior to the registration of the participant for the trial and collection of baseline data. For participants who score ≤ 18/30 on the MoCA during the interview, written consent will also be obtained from the accompanying/nominated relative, friend or whanau.

### Confidentiality

Participants’ records and the data generated by the study will be confidential in line with the recommendations of the Health Research Council and The Health Information Privacy Code 1994 (HIPC) privacy legislation. Data will be stored in a secure, lockable location. Electronic data storage will be password protected. Standardised electronic case report forms (CRFs) will be provided for each participant enrolled in this study. The participants in this study are identified only by participant identifier number on these forms. Any information that may identify a participant will be excluded from data presented in the public arena.

### Dissemination

Participants and their family/close friends will be invited to annual dissemination meetings. Results presented vary with the study phase from recruitment progress (during the recruitment phase), participants’ characteristics (during the follow-up phase) and findings about the main research question after data collection is completed and analysed. Study results will also be provided to the local medical community, healthcare professionals and health service providers through seminars. Mainstream and social media will be engaged to disseminate results to the wider public community. For the academic community, results will be published in peer-reviewed journals and presented in conferences.

### Pilot study

A pilot study was carried out between 2014 and 2015 to assess the feasibility of this randomised controlled trial. The pilot study aimed to identify feasibility of the recruitment strategies and the outcome measures, implementation of the SC and SAYGO programmes, and to refine the processes. The pilot study was completed in two of the four sites similar to the main study (Whangarei and Howick) as these locations are ‘new territory’ for research with older adults; the other two have established relationships with local stakeholders. We aimed to recruit 80 older adults with similar eligibility criteria as mentioned above.

We invited 527 age-eligible older adults from four general practices through regular post; 433 (82%) responded to the invitation. Of this, 107 (25%) were interested and eligible; 71/107 were enrolled in the pilot study.

The randomisation procedure was implemented effectively and the utility of the outcome measures is found to be good. The intervention programmes were initiated successfully but the transitioning period to a peer-led class required a longer duration compared to existing community classes. This was attributed to the frailer health status of the sample.

Overall, the pilot study demonstrated that the recruitment strategies were acceptable and additional resources (facilitator time) are required for successful transition to peer-led classes. Three changes were made to the main study: (1) switching from MMSE to MoCA due to MMSE copyright issues; (2) adjusting the eligible age to 60+ (from 75+) for Pasifika as they have similar health profile to Māori; and (3) switching from paper-based questionnaire to the web-based EDC to streamline the data entry process.

## Discussion

Older people aged 75+ are often being excluded from clinical trials due to heterogeneity of the group. With the growing ageing population, it is no longer acceptable to extrapolate findings from younger populations. Evidence from the older adults’ population is needed to inform best practice that could be offered by health professionals.

The prevalence of frailty increases with ageing. Studies showed frailty is related to disability, adverse health outcomes and increased health care utilisation [3, 52, 53]. Recognising this demographic shift and the prediction of a higher burden on the healthcare system, ongoing initiatives around the world are examining the effectiveness of multicomponent interventions to maintain and improve function and quality of life [54, 55] which are valued by older people [56], and payers are exploring cost effective strategies to facilitate ageing in place.

The Senior Chef and SAYGO programmes are well accepted by community-living older adults aged 65+ (≥ 55 years for Māori and Pacific people). Evaluation on the long-term (12 months) impact of these programme is promising [12, 22–24]. In the SC programme, there was a significant increase in cooking skills, confidence in cooking, food and nutrition knowledge, and intake of milk and milk products 12 months post-course [12]. Similar trend was observed in the SAYGo programme. Improvement on functional measures of gait and balance observed after the 10-week persisted at 12-month follow-up [22]. Participants also found camaraderie in these programmes and increased social networks [12, 24]. We speculate that most of these older adults are robust—suggested by self-referral and were of younger age group. These programmes demonstrate potential to benefit pre-frail older adults. Any study working with older people and their usual health providers is complex in that older people require additional time, explanation and engagement [57] and working with existing health providers present challenges in recruitment. Success in past primary care trials has shown this is feasible in the New Zealand context [58].

This trial is designed to be a real-world rigorous assessment to address two main questions: are nutrition and/or strength and balancing exercise programmes effective in preventing frailty in pre-frail community-dwelling older people? If yes, how cost effective are these programmes? The first question helps public health professionals to understand which strategy is better in postponing frailty and the process outcomes facilitate the understanding of the underlying changes prior to observable clinical improvements. It is also the interest of older adults to maintain function and independence in later years. The second question is a fundamental interest to payers and policy-makers. We also evaluate the sustainability of participation after the formal facilitated programmes with an opportunity to enhance the role of older people through peer-led intervention. We aim for long-term follow-up to 24 months. Recruitment to the current study began in April 2016 and the trial expects to report in 2020.

We acknowledge the importance of working in partnership with the older adults and the local communities to facilitate sustainability of the intervention programmes. Feedback will be sought from the study participants and the programme facilitators to support the transition into peer-led groups. The health approach of older Māori and Pasifika is likely to be unique. It is vital for us to work with the relevant local stakeholders to guide the adaptation of the intervention programme to ensure these are culturally appropriate and acceptable. Consultations with relevant groups and older Māori/Pasifika are carried out prior to the initiation of the trial.

If successful, this will generate valuable information about efficacy of the nutrition and exercise strategies, but also provide insightful information for implementation. We will report exactly what happened and involvement of the participants and stakeholders in this trial. Trials require replication as strategies may have different impacts in different health systems and populations, but without well-designed and rigorously conducted trial set in the real world, attempts to reduce frailty and improve outcomes for older people in our ageing societies may waste valuable resources.

## Trial status

Protocol Version 3 dated 26 February 2016. The recruitment was initiated in April 2016 and first participant enrolled on 12 May 2016.

## Data Availability

The datasets generated and/or analysed during the current study are available from the corresponding author on reasonable request.
