# Correction to: Study protocol of a randomised controlled trial to examine the impact of a complex intervention in pre-frail older adults

**DOI:** 10.1007/s40520-022-02162-4

**Published:** 2022-06-16

**Authors:** Ruth Teh, Ngaire Kerse, Debra L. Waters, Leigh Hale, Avinesh Pillai, Evelingi Leilua, Esther Tay, Anna Rolleston, Richard Edlin, Eruera Maxted, Claire Heppenstall, Martin J. Connolly

**Affiliations:** 1 Department of General Practice and Primary Health Care, School of Population Health, University of Auckland, Auckland, New Zealand; 2 School of Population Health, University of Auckland, Auckland, New Zealand; 3 Department of Medicine, School of Physiotherapy, University of Otago, Dunedin, New Zealand; 4 Centre for Health, Activity and Rehabilitation Research, School of Physiotherapy, University of Otago, Dunedin, New Zealand; 5 Department of Statistics, Faculty of Science, University of Auckland, Auckland, New Zealand; 6 The Centre of Health, Tauranga, New Zealand; 7 Health Systems Group, School of Population Health, University of Auckland, Auckland, New Zealand; 8 Lakes District Health Board, Rotorua, New Zealand; 9 Department of Medicine, University of Otago, Christchurch, New Zealand; 10 Department of Geriatric Medicine, University of Auckland, Auckland, New Zealand; 11 Waitemata District Health Board, Auckland, New Zealand

## Correction to: Aging Clinical and Experimental Research (2019) 31:1407–1417 https://doi.org/10.1007/s40520-018-1106-7

The article Study protocol of a randomised controlled trial to examine the impact of a complex intervention in pre-frail older adults, written by Ruth Teh, Ngaire Kerse, Debra L. Waters, Leigh Hale, Avinesh Pillai, Evelingi Leilua, Esther Tay, Anna Rolleston, Richard Edlin, Eruera Maxted, Claire Heppenstall and Martin J. Connolly was originally published electronically on the publisher’s internet portal (currently SpringerLink) on 02 January 2019 without open access. With the author(s)’ decision to opt for Open Choice the copyright of the article changed on 25 May 2022 to © The Author(s) 2022 and the article is forthwith distributed under the terms of the Creative Commons Attribution 4.0 International License (http://creativecommons.org/licenses/by/4.0/), which permits use, duplication, adaptation, distribution and reproduction in any medium or format, as long as you give appropriate credit to the original author(s) and the source, provide a link to the Creative Commons license and indicate if changes were made.

The original article has been corrected.

